# Case of Poison from a Vegetable Fungus; Together with Remarks on Professor Rossi's Two Cases of Supposed Rabies Canina

**Published:** 1804-12-01

**Authors:** Samuel Argent Bardsley

**Affiliations:** Physician to the Manchester Infirmary


### Case of Poison from a Vegetable Fungus; *together with Remarks on Professor* Rossi's *two Cases of* supposed *Rabies Canina; by* Samuel Argent Bardsley, M.D. *Physician to the Manchester Infirmary.*

( Continued from our last, pp. 385—391. )

TWO or three days after the accident,* I requested Dr. Hull, (whose botanical knowledge is well known, and justly appreciated) to accompany me with my patient, and his companion, to the spot, where they had gathered and eaten the fungus. We found, in different states of growth, a species of agaric, which the children immediately pointed out as the one they had partaken of. This species is certainly different from any of those enumerated as poisonous by Plenk and Balliard.

The *agaricus bulbosus,* of Sowerby, (plate 130) approaches the nearest to it of any which Dr. Hull has ever seen. The sensible qualities were not such as would lead one to suspect its poisonous nature. Its juice was neither milky nor acid. In smell and taste it was not very unlike the common mushroom, or the kernel of an unripe hazel nut.

The following description of this agaric will perhaps enable some botanical reader to decide, whether the fungus be an undescribed species, and may tend to prevent the bad consequences arising from eating it in future.

### *Description of the Agaric.*

Stalk central, solid, bulbous at the base, gradually attenuated upwards, curved, ascending; brownish buff; length from three to four inches; diameter at the base from half to three-quarters of an inch; ring cobweb-like, or wanting; no wrapper.

Pileus brown buff, darker in the centre; somewhat convex, slightly bossed, margin turned in; diameter from one inch and a half to four inches.

Gills buff, somewhat decurrent, giving a scored appearance to the stalk, as low as the ring, or remains of the curtain; very numerous, four in each series, two of the

* These additional Observations of Dr. Bardsley were intended by him to have been given in his Paper which appeared in the last month's Journal; but from accident, they were not received until his first Communication had been sent to press. Edit.

the loose gills very small, the middle one extending more than half way to the stalk.

---

The second case of *supposed* canine madness, recorded by Prof. Rossi, is scarcely deserving any comment. No one symptom characteristic of the disease is stated to have made its appearance. The patient had been bitten by a dog, said to be rabid; and the uneasiness and dread of danger arising from this accident, naturally deprived him of sleep. Opium, instead of alleviating, seemed rather to increase his restlessness. We are indeed told, in vague terms, "that the patient even began to feel an obstruction in his throat;" and that immediately upon this symptom appearing, Prof. Rossi commenced his Galvanic operations, and in fifteen days the patient recovered. Of what did he recover? Surely no one will reply, "of *rabid hydrophobia*." The idea is too absurd to be entertained for a single moment. I mean not by these remarks to deny that Galvanism may possess a salutary influence, in subduing many obstinate diseases. Its merits as a remedy cannot, at present, be duly appreciated. It would appear from Prof. Rossi's representation, that it acts powerfully on the nervous system, and seems likely to prove an useful auxillary in the cure of several diseases, arising from inordinate spasmodic action. But that it is capable of subduing *genuine hydrophobia*,* remains yet (unfortunately for mankind!) to be verified by experiments, conducted with impartiality, ability, and accuracy.

*Manchester, October* 11, 1804.

---

### To Dr. GILLESPIE.

Sir,

I Take the liberty of sending you three cases, which I request you will have the goodness to put into a proper form, and if advisable, to publish them through the medium of your learned and respectable friend, Dr. S. Should

---

* I intend soon to publish the History of a fatal Case of Hydrophobia, with the appearances upon dissection, (the case I have already alluded to) in which it will appear, that *Galvanic shocks* were tried *in vain*; together with the exhibition of large doses of the volatile caustic alkali, and cantharides, in substance.

